# Intelligent Inspection Method and System of Plastic Gear Surface Defects Based on Adaptive Sample Weighting Deep Learning Model

**DOI:** 10.3390/s24144660

**Published:** 2024-07-18

**Authors:** Zhaoyao Shi, Yiming Fang, Huixu Song

**Affiliations:** Beijing Engineering Research Center of Precision Measurement Technology and Instruments, College of Mechanical & Energy Engineering, Beijing University of Technology, Beijing 100124, China; fangandyim@163.com (Y.F.); huixu_song@bjut.edu.cn (H.S.)

**Keywords:** surface defect inspection, deep learning, adaptive sample weighting, automatic control system, plastic gears

## Abstract

After injection molding, plastic gears often exhibit surface defects, including those on end faces and tooth surfaces. These defects encompass a wide range of types and possess complex characteristics, which pose challenges for inspection. Current visual inspection systems for plastic gears suffer from limitations such as single-category defect inspection and low accuracy. There is an urgent industry need for a comprehensive and accurate method and system for inspecting defects on plastic gears, with improved inspection capability and higher accuracy. This paper presents an intelligent inspection algorithm network for plastic gear defects (PGD-net), which effectively captures subtle defect features at arbitrary locations on the surface compared to other models. An adaptive sample weighting method is proposed and integrated into an improved Focal-IoU loss function to address the issue of low inspection accuracy caused by imbalanced defect dataset distributions, thus enhancing the regression accuracy for difficult defect categories. CoordConv layers are incorporated into each inspection head to improve the model’s generalization capability. Furthermore, a dataset of plastic gear surface defects comprising 16 types of defects is constructed, and our algorithm is trained and tested on this dataset. The PGD-net achieves a comprehensive mean average precision (mAP) value of 95.6% for the 16 defect types. Additionally, an online inspection system is developed based on the PGD-net algorithm, which can be integrated with plastic gear production lines to achieve online full inspection and automatic sorting of plastic gear defects. The entire system has been successfully applied in plastic gear production lines, conducting daily inspections of over 60,000 gears.

## 1. Introduction

**Figure 1:**
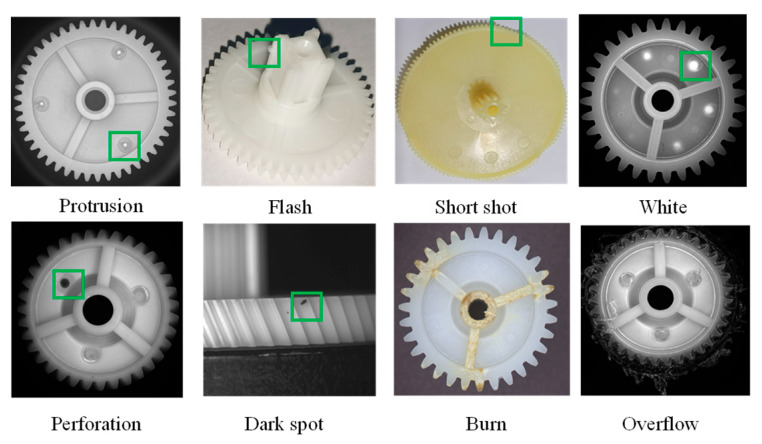
Partial surface defects of plastic gears. The green box marks the location of the defect.

During the manufacturing process, plastic gears can develop a wide variety of surface defects due to issues related to materials, equipment, and processes [1,2]. As illustrated in Figure 1, common defects include flash, dark spots, void, point gate perforation, point gate protrusion, white, overflow, and burn, occurring on both end faces and tooth surfaces. Based on the causes of defect formation, they can be categorized into the following four types:

(1) Injection molding defects caused by the molding process, such as flow marks, white, and burn;
(2) Injection defects related to the storage or use of materials, including color variations and foreign particle inclusions (such as dark spots);
(3) Injection defects caused by maintenance issues or poor mold design, such as flash, bubbles, overflow, oil contamination, and short shot;
(4) Defects resulting from improper human operations, such as scratches from manually removing flash and tooth surface damage from mishandling.

In response to the vast production volume and diverse surface defects of plastic gears, there is a pressing need within the plastic gear industry for an online automated inspection device, as traditional gear measuring instruments fail to meet this demand. Therefore, visual online inspection technology for gears has rapidly developed, offering advantages such as non-contact operation, high inspection efficiency, and comprehensive information acquisition, which facilitates integration with automated equipment to achieve online inspection of plastic gear defects [3,4,5].

Traditional defect inspection algorithms often struggle to accurately distinguish the complex surface defect features of plastic gears. With the rapid development of deep learning technology, defect inspection algorithms based on deep learning have shown significant improvements in inspecting multiple complex defects. However, challenges still exist, such as the real-time problem, small sample problem, small target problem, and unbalanced sample problem [6,7,8,9,10,11,12,13].

Sun et al. [14] proposed a cascade inspection method for surface defects based on high-resolution inspection images. By introducing Euclidean distance width deviation in the CIoU localization loss function, they effectively inspected wireframe defects of multiple scales, achieving *mAP* of 85.01% and an inference time of 37 ms. The method attained inspection accuracy exceeding 95%, with a false negative rate controlled below 6%, but only inspected three types of defects: dirt, white scratches, and black scratches. He et al. [15] introduced a novel steel plate defect inspection system. The system employed a baseline convolutional neural network to generate feature maps at each stage, and a multi-layer feature fusion network (MFN) to merge multiple hierarchical features into one, enhancing the feature’s ability to capture position details of more defects. The study established the NEU-DET defect inspection dataset, demonstrating accuracy rates of 99.67% for defect classification and 82.3% *mAP* for defect inspection. However, the system could only inspect six types of steel plate defects.

In recent years, some scholars have conducted research on gear defect inspection algorithms in the field of deep learning. Zhang et al. [16] proposed an improved YOLO-v3 network to inspect stain and miss defects in plastic gears, achieving a false inspection rate of 1.3%. However, this study only considered two types of end-face defects and did not address tooth surface defects. Kamal et al. [17] employed two classification methods based on convolutional neural networks to identify scratches, protrusions, hole erosion, and asymmetric block defects in gears. The first method had a shorter processing time of 0.09 s, with an accuracy of 92%, while the second method achieved a higher accuracy of 96.5% with an average time of 0.67 s. Nonetheless, the study focused on inspecting defects in metal gears, which has unknown effectiveness for plastic gear inspection. Xi et al. [18] developed an integrated Yolov5-Deeplabv3+ real-time segmentation network for online measurement of pitting defects in metal gears, addressing sample imbalance issues. However, the study only considered one type of pitting defect in metal gears and lacked experimental results for mixed defect inspection. Xiao et al. [19] designed an improved GA-PSO algorithm for identifying four types of defects in powder metallurgy gears: tooth breakage, wear, cracking, and scratching, achieving an accuracy rate of over 94%. However, tooth surface defect inspection was not addressed.

In summary, existing deep learning algorithms for gear defect inspection still face challenges such as limited defect inspection types, low accuracy, and insufficient research on tooth surface defect inspection.

Furthermore, apart from defect inspection algorithms, it is equally important to design the structure and control system of a gear defect inspection system. Most defect inspection systems are unsuitable for adopting vibration plate feeding methods [20,21], which will cause significant damage to plastic gears. Gear defect inspection systems can be classified into two types: integrated on conveyors and independently mounted on glass turntables. Reference [22] describes gear visual online inspection devices located above conveyors, which occupy less space, but have many factors resulting in low inspection accuracy, e.g., unstable conveyor motion, susceptibility to wear, and variable product placement angles. Additionally, the non-transparency of conveyors prevents image capture of the gear bottom surface. Reference [23] introduces a glass turntable-based injection-molded gear online inspection and sorting system, for inspecting surface defects such as dark spots and flash without flipping the product to obtain bottom-side inspection images. However, the device inspects fewer types of defects and cannot collect tooth surface defect data.

All in all, plastic gear defect online inspection systems face three main challenges: First, the diversity and complexity of plastic gear defects, encompassing both end-face and tooth surface defects, pose significant inspection difficulties, with a lack of research on specialized multi-class defect fusion inspection algorithms for plastic gears. Second, research on plastic gear defect online inspection devices is scarce, so that the industry lacks practical solutions for coupling online inspection equipment with plastic gear production lines. Third, it poses significant challenges to ensure the normal operation and coordination of various modules because integrating defect inspection algorithms with automated inspection equipment requires comprehensive consideration of mechanical structure, control systems, and inspection algorithms, involving multiple complex technologies [24].

To address the above issues, this study developed a method and system for inspecting comprehensive surface defects on plastic gears, enabling intelligent online inspection of 16 types of surface defects on plastic gears. The system has been successfully deployed in production lines. In the following, we will discuss it point by point.

## 2. Plastic Gear Online Inspection System

**Figure 2:**
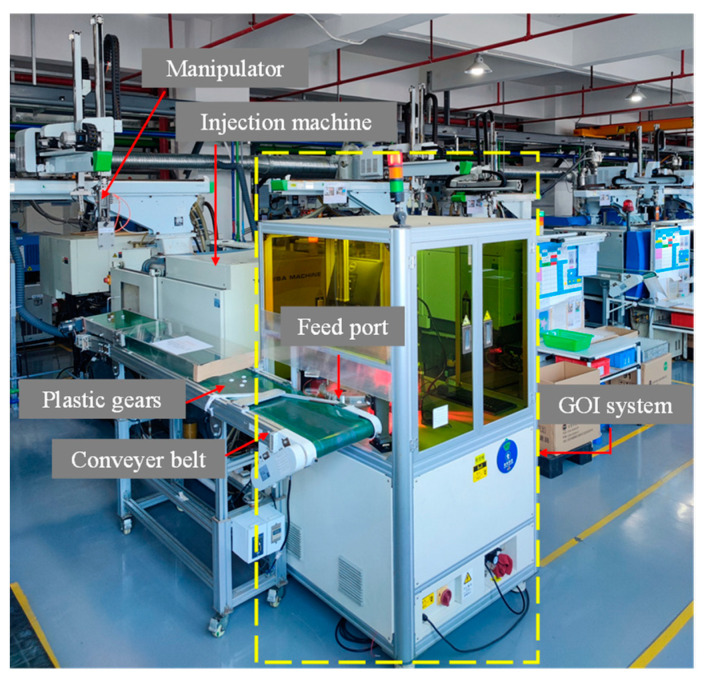
GOI system on plastic gear production line.

The gear online inspection system (GOI system) developed in this study has been successfully deployed on plastic gear production lines, tightly integrated with the assembly line to achieve real-time online inspection and sorting of plastic gears. Over 60,000 plastic gears are inspected daily, with a physical representation of the production line shown in Figure 2.

**Figure 3:**
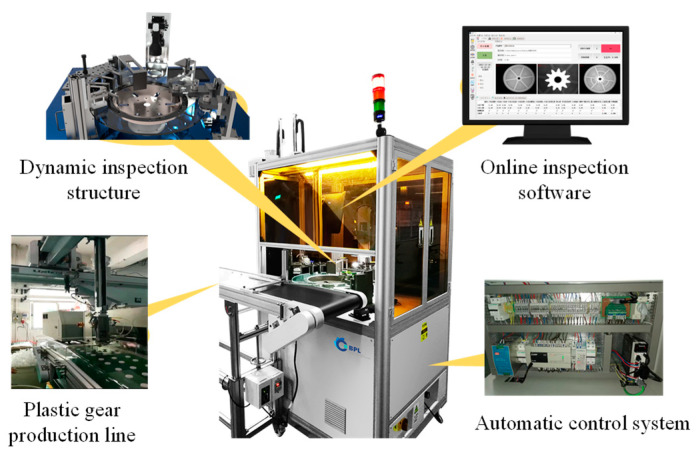
Composition of GOI system.

The composition structure of the GOI system is illustrated in Figure 3, consisting of three main components: the dynamic inspection structure, the automatic control system, and the online inspection software.

### 2.1. Dynamic Inspection Structure

**Figure 4:**
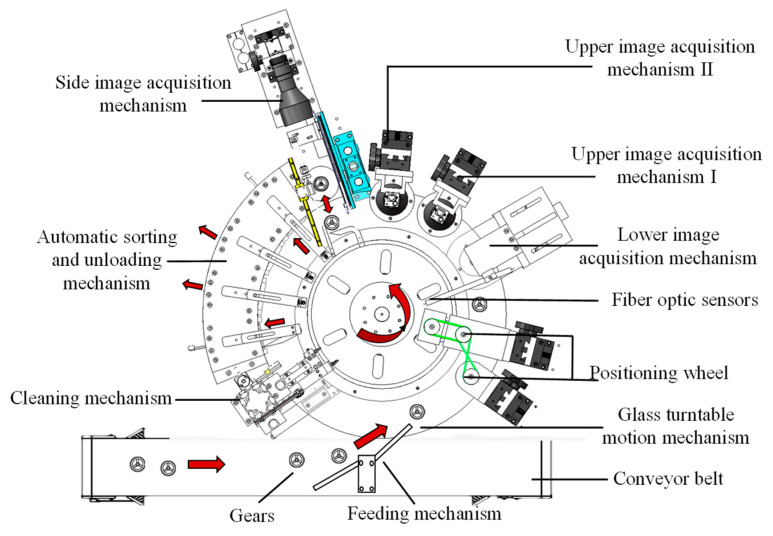
Composition of the dynamic inspection structure. The red arrow indicates the direction of motion of the gears.

As shown in Figure 4, the dynamic inspection mechanism consists of several components, including the feeding mechanism, glass turntable motion mechanism, radial positioning wheel, fiber optic sensor, lower image acquisition mechanism, upper image acquisition mechanism I, upper image acquisition mechanism II, side image acquisition mechanism, automatic sorting and unloading mechanism, and cleaning mechanism. The entire process of plastic gear inspection and sorting is completed under dynamic rotation.

Firstly, a feeding mechanism is designed to achieve non-manipulator feeding. The glass turntable rotates counterclockwise along the axis under servo motor control, and the processes of gear feeding, positioning, image acquisition, and sorting and unloading are all driven by the glass turntable motion mechanism. Other mechanisms are arranged around the turntable in the order in which the gears pass. The gears are clamped within a fixed radius range by the radial positioning wheel, ensuring they are within the camera’s field of view. The fiber optic sensor automatically inspects the signal when a gear arrives and can determine its circumferential position. The lower image acquisition mechanism, upper image acquisition mechanisms I and II, and side image acquisition mechanism are used to capture images of the bottom end face, upper end face (small tooth), upper end face (large tooth), and tooth surface of the plastic gears, respectively, achieving multi-surface image acquisition of the gears. The cleaning mechanism is used to clean the surface of the glass turntable, removing any interfering stains.

**Figure 5:**
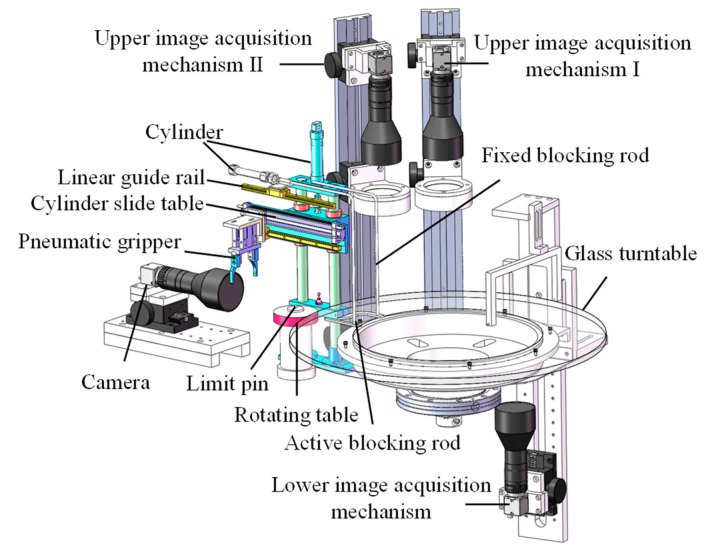
Full surface image acquisition mechanism.

In order to automatically capture images of the tooth surface around the circumference of the plastic gear, a special design was implemented for the side image acquisition mechanism, which differs significantly from the other three image acquisition mechanisms. As illustrated in Figure 5, when the plastic gear reaches the side image acquisition mechanism, it is immediately inspected. At this point, the cylinder and slide table act together, causing the active blocking rod to move in the direction away from the axis. This, in conjunction with the fixed blocking rod, halts the gear’s movement. Subsequently, the pneumatic gripper clamps the gear and places it on the rotating table. The center of the rotating table is aligned with the center hole of the gear by a limit pin, securing the gear in place. The built-in motor within the rotating table rotates the gear one full revolution, while the side-mounted camera rapidly captures multiple tooth surface images covering the circumference of the gear, based on a predetermined frame rate.

### 2.2. Automatic Control System

**Figure 6:**
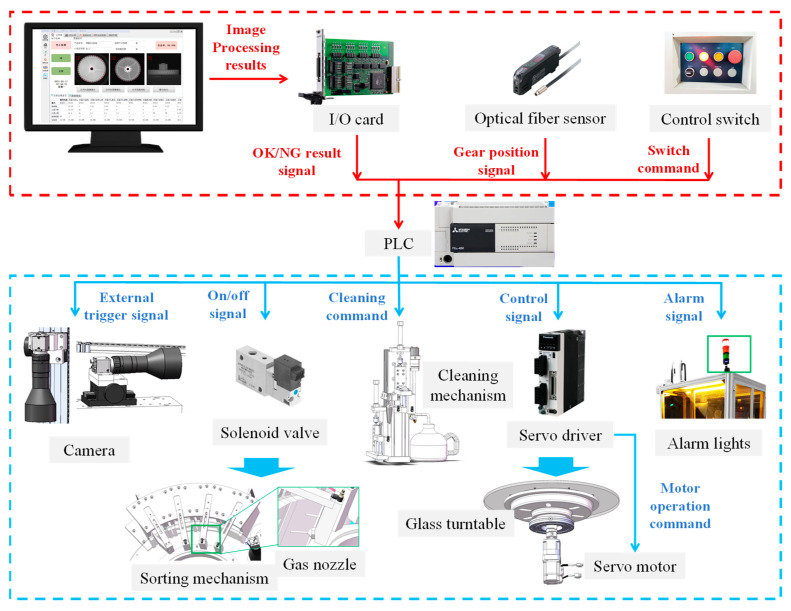
Composition of automatic control system.

The automatic control system of the GOI system is depicted in Figure 6, where a PLC serves as the central processor. It comprises input control modules, sensors, and actuating components, aimed at achieving motion control, positioning, image acquisition, inspection, sorting and unloading, as well as anomaly alert functionalities.

**Figure 7:**
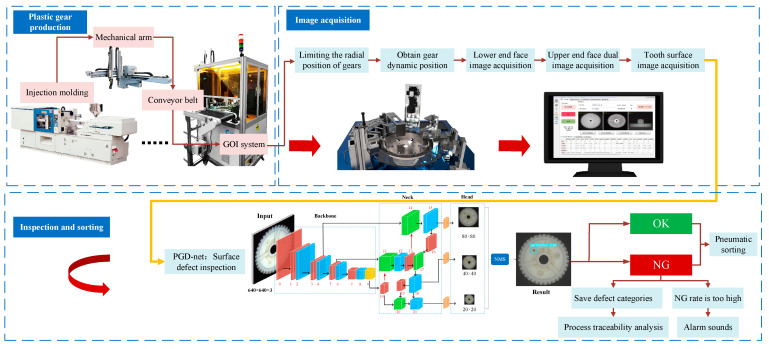
Automatic inspection flowchart.

Referring to Figure 7, after the plastic gear is demolded, it is placed onto the conveyor belt by a robotic arm. The gear is then conveyed to the glass turntable for loading via the feeding mechanism. The PLC controls the servo motor to rotate the glass turntable counterclockwise starting from the loading position of the gear. When the gear reaches each image acquisition mechanism, the PLC triggers the corresponding camera to capture an image of the gear. Particularly, when the gear reaches the side image acquisition mechanism, the system first controls the pneumatic gripper to place the gear onto the rotating table. Subsequently, multiple images covering the circumference of the gear are captured. The online inspection software processes the gear images to produce inspection results, which are then sent to the PLC. When the gear reaches the corresponding discharge chute, the PLC sends an on/off signal to the respective solenoid valve, causing the air nozzle at the entrance of the chute to release air momentarily as the gear passes through, thereby blowing the gear into the corresponding discharge chute. This completes the entire process of plastic gear inspection and sorting.

## 3. Intelligent Inspection Network for Comprehensive Defects of Plastic Gears: PGD-net

At the core of the GOI system lies the online inspection software. We collected and established a dataset of surface defects on the upper end face (ejector pin point face), lower end face (gate point face), and side face (entire tooth surface) of plastic gears. Based on the sample distribution characteristics of the dataset, we constructed a deep learning network called Plastic Gear Defect-net (PGD-net). The basic architecture of this network model is based on YOLOv5’s Backbone, Neck, and Head, and incorporates a new sample weight adaptive optimization method. It outperforms YOLOv5 in terms of sample balance, inference speed, and generalization ability. The online inspection software integrated into PGD-net can accurately and rapidly identify 16 types of surface defects from the multi-surface images of plastic gears captured.

### 3.1. PGD-net Architecture

**Figure 8:**
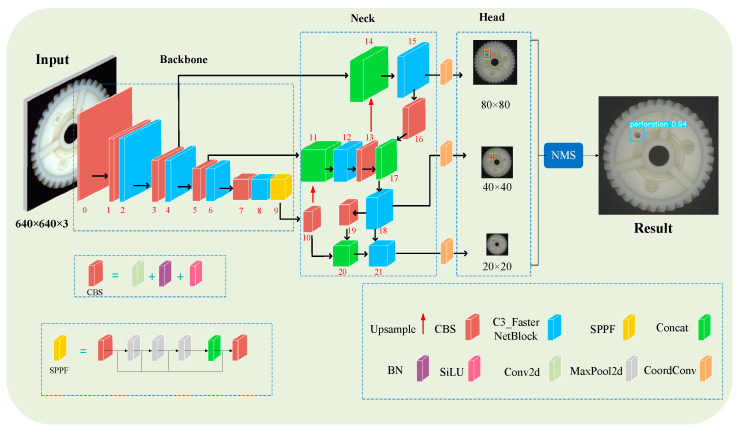
PGD-net architecture.

As shown in Figure 8, the structure of PGD-net consists of three classic parts: Backbone, Neck, and Head.

Firstly, the Backbone is connected to the input end and is responsible for extracting features from input images, with the specified input image size being 640 × 640. The Backbone and Neck together comprise 10 Convolution-BatchNorm-SiLU (CBS) modules. Each CBS module consists of a convolutional layer, a Batch Normalization (BN) layer, and a *SiLU* layer (an activation function). The equation for the *SiLU* function is:

(1) $$SiLU(x)=x\cdot Sigmoid(x)=x/(1+e^{-x})$$

**Figure 9:**
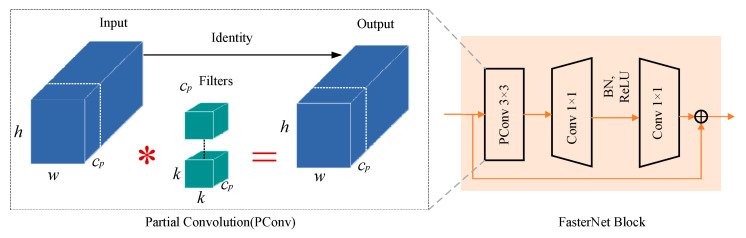
FasterNet Block.

The Backbone and Neck consist of a total of eight C3-FasterNetBlock modules. In this study, all regular convolutional layers in the original C3 modules were replaced with FasterNet Blocks [25]. The structure of the FasterNet Block, as shown in Figure 9, is utilized to reduce redundant computations and memory access, effectively extracting spatial features.

**Figure 10:**
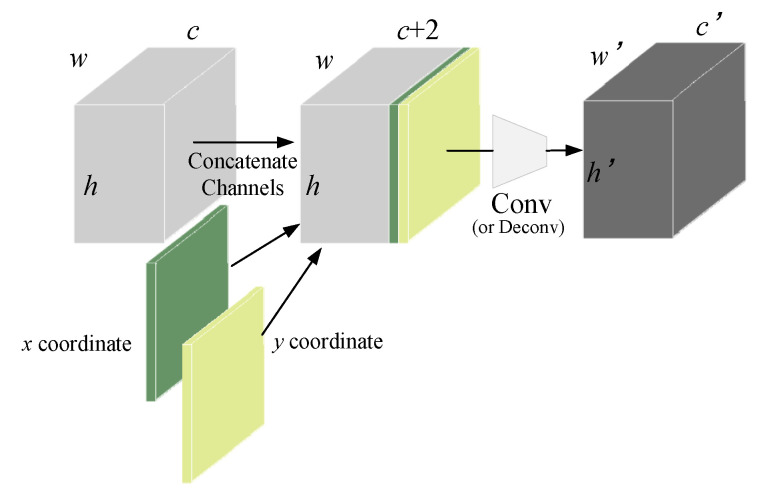
CoordConv Layer.

In this study, CoordConv [26] was incorporated into each inspection head in the Head, as illustrated in Figure 10. Compared to traditional convolution, CoordConv simply adds two additional channels behind the input feature map, one representing the x-coordinate and the other representing the y-coordinate, while leaving the rest unchanged. Traditional convolution possesses three characteristics: few parameters, computational efficiency, and translational invariance. However, CoordConv inherits only the first two characteristics, allowing the network to maintain or discard translational invariance based on its learning process. While it may seem that this would impair the model’s generalization ability, dedicating a portion of the network’s capacity to model non-translational invariance can actually enhance the model’s generalization capability.

### 3.2. Adaptive Sample Weighting Method: Focal-IoU Loss

In object inspection, the Intersection over Union (IoU) value represents the ratio of the intersection and union between the predicted box A and the ground truth box B, as follows:

(2) $$IoU=\frac{\left(A\cap B\right)}{\left(A\cup B\right)}$$

**Figure 11:**
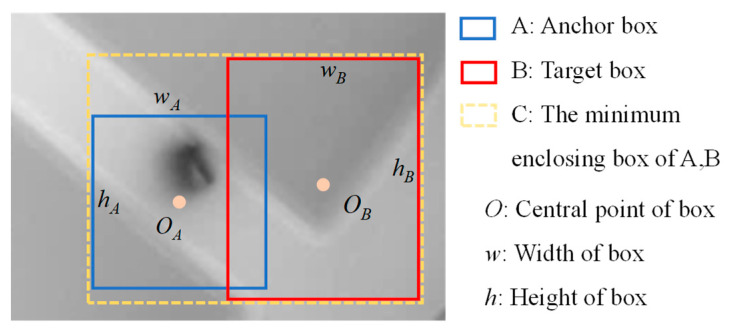
The geometric relationship between IoU boxes.

The IoU loss function [27], widely used in object inspection tasks, directly optimizes the IoU value. However, it may not precisely reflect the overlap between the predicted box and the ground truth box. Therefore, several improved versions have been proposed, including the GIoU [28], DIoU, and CIoU [29] loss functions, as depicted in Figure 11.

The definition of GIoU is as follows:

(3) $$L_{GIOU}=1-IOU+\frac{\left|C-(A\cup B)\right|}{\left|C\right|}$$

where |C| represents the area of the minimum convex bounding box containing both A and B. The disadvantage of GIoU is that it tends to produce a larger bounding box when the two boxes are far apart, and it has a slow convergence speed.

Addressing the drawbacks of GIoU, DIoU offers improvements. Its equation is as follows:

(4) $$L_{DIoU}=1-IoU+\frac{\rho^{2}(O_{A},O_{B})}{d^{2}}$$

In the equation, $\rho (O_{A},O_{B})$ represents the Euclidean distance between the center points of boxes A and B, while *d* is the diagonal distance of the minimum enclosing box around them.

CIoU, based on DIoU, takes into account both the center point distance and aspect ratio. It is defined as:

(5) $$L_{CIoU}=1-IoU+\frac{\rho^{2}(O_{A},O_{B})}{c^{2}}+\alpha \nu$$

where α is a weighting function and ν is used to measure the consistency of aspect ratios, defined as follows:

(6) $$\alpha =\frac{\nu }{(1-IoU)+\nu }$$

(7) $$\nu =\frac{4}{\pi^{2}}{\left(\arctan\frac{w_{B}}{h_{B}}-\arctan\frac{w_{A}}{h_{A}}\right)}^{2}$$

Among them, $w_{A}$ and $h_{A}$ represent the width and height of predicted box A. $w_{B}$ and $h_{B}$ represent the width and height of ground truth box B.

However, the vast majority of plastic gear products in daily production are qualified, resulting in a scarcity of defective samples. Additionally, there exists a long-tail effect in the distribution of different types of defect samples, leading to severe class imbalance in the sample distribution, which significantly affects model convergence. To address this issue, this study incorporates the idea of focal loss [30] into the IoU loss function. This involves adapting the contribution of samples to the loss based on their prediction accuracy, aiming to enhance the loss of hard samples and facilitate the exploration of difficult samples. However, the specific calculation formula is different from the existing focal loss methods.

The specific approach is as follows: we introduce an adjustment factor γ for sample difficulty into GIoU, DIoU, and CIoU, respectively, to reduce the impact of easy-to-classify samples. The equations are as follows:

(8) $$L_{Focal-GIoU}={\left(1-IoU\right)}^{\gamma }\cdot L_{GIoU}$$

(9) $$L_{Focal-DIoU}={\left(1-IoU\right)}^{\gamma }\cdot L_{DIoU}$$

(10) $$L_{Focal-CIoU}={\left(1-IoU\right)}^{\gamma }\cdot L_{CIoU}$$

Here, a higher IoU indicates that the sample is easier to inspect, while a lower IoU indicates that the sample is more challenging to inspect. Therefore, when γ>0, the loss for samples with higher IoU will be smaller, causing the weight of easy-to-inspect samples to decrease, while the weight of hard-to-inspect samples will increase. This acts as a weighting mechanism, assigning a larger loss to more challenging targets, thereby helping to improve the regression accuracy of difficult samples.

## 4. Experimental Results and Discussion

### 4.1. Experimental Condition

This study builds a deep learning framework based on PyTorch 2.0 on a Windows 10 system. The GPU used is an NVIDIA GeForce GTX 4090 with CUDA version 11.8 and 24 GB of memory. The CPU is an Intel Core i9-13900K. The hardware parameters of the visual inspection system are shown in Table 1.

**Figure 12:**
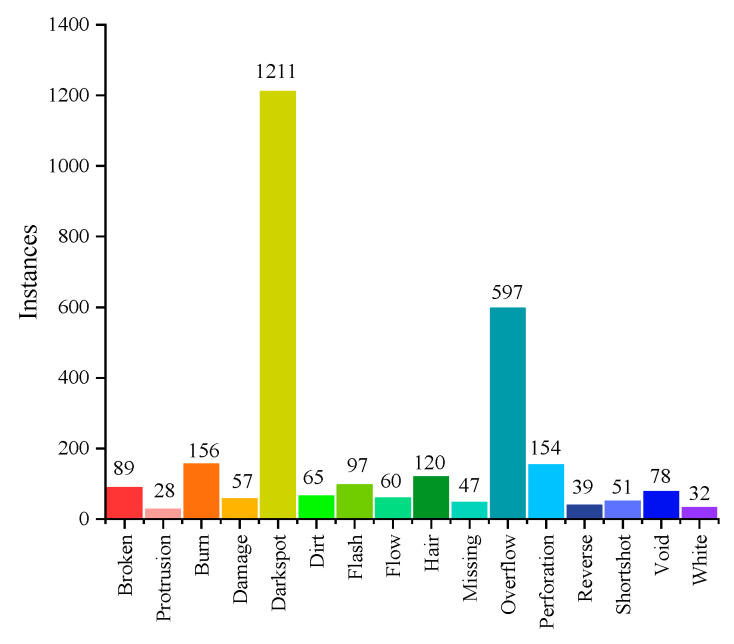
Statistics on the number of 16 defect samples in the training set.

A dataset of plastic gear surface defects was established, comprising a total of 922 end-face images and 518 tooth surface images, with a total of 16 defect types and a total of 2881 samples. The dataset was split into training, validation, and test sets in an 8:1:1 ratio. The number of instances for each defect in the training set is shown in Figure 12. The original image resolution is 2048 × 2448.

### 4.2. Evaluation Indicators

We evaluated the performance of the model using recall (*R*), accuracy (*P*), mean precision (*AP*), mean average precision (*mAP*), *F*1-score, and inference speed, respectively.

The equation for calculating the recall rate *R* is as follows:

(11) $$R=\frac{TP}{TP+FN}$$

In the equation, *TP*, *TN*, *FN*, and *FP* represent the number of samples with true positive, true negative, false negative, and false positive, respectively.

The equation for calculating accuracy *P* is as follows:

(12) $$P=\frac{TP}{TP+FP}$$

The mean precision (*AP*) is the area calculated based on the P–R Curve (Precision–Recall Curve), which is used to comprehensively consider the performance of the model under different recall rates. The value range of *AP* is between 0 and 1, and a higher value indicates better performance of the model in inspecting target instances. The calculation equation is as follows:

(13) $$AP=\int_{0}^{1}p(r)dr$$

But usually, approximation or difference methods are used to calculate *AP*, and the equation is as follows:

(14) $$AP=\sum_{k=1}^{N}P(n)\Delta R(n)$$

In the equation, *N* is the total amount of data, and *n* is the index of each sample point.

(15) ΔR(n)=R(n)−R(n−1)

Mean Average Precision (*mAP*) is the average *AP* value of all categories, calculated as follows:

(16) $$mAP=\frac{\sum_{i=1}^{k}AP_{i}}{k}$$

In the equation, *k* is the number of categories.

*mAP:0.5* refers to the *mAP* calculated using an *IoU* threshold of 0.5 in object inspection tasks.

*mAP:0.5,0.95* refers to the *mAP* calculated at Intersection over Union (IoU) thresholds of 0.5 and 0.95, respectively.

*F*1-score is the harmonic mean of Precision and Recall, used to comprehensively assess the accuracy and recall capabilities of an algorithm. The calculation equation is as follows:

(17) $$F1=\frac{2P\cdot R}{P+R}$$

### 4.3. PGD-net Inspection Performance Experiment

To evaluate the inspection performance of PGD-net, this study compared it with current mainstream object inspection models, and the results are shown in Table 2. After training for 300 epochs, PGD-net performed the best on metrics such as *mAP:0.5*, Precision, Recall, False Positive Rate (FPR), and False Negative Rate (FNR). It achieved an *mAP:0.5* of 95.6%, which is 2.5% higher than the second-ranked YOLOv5 model. The FPR and FNR of PGD-net were also the lowest among all models, at 0.2% and 6.7%, respectively, which are crucial for ensuring product quality. Besides demonstrating excellent inspection performance, PGD-net also showed decent training speed, reaching 21 ms/piece, and inference speed of 44.2 ms/piece. All inference was conducted on the CPU, and if GPU inference is used, the average speed of PGD-net can reach 4.3 ms/piece.

Table 2 reflects the overall inspection performance of each model for all defect categories.

Before further analysis, to provide readers with a more intuitive understanding of the 16 types of surface defects inspected by the models, we first present images of the 16 defects in Table 3 and provide explanations.

In order to reflect the individual inspection capability of each model for each defect, we compiled the inspection results of various models for the 16 types of surface defects on plastic gears, using *mAP:0.5* as the metric, and summarized them in Table 4. Analysis of Table 4 reveals that defects such as Broken, Protrusion, Missing, Overflow, Perforation, Reverse, Short Shot, and White are relatively easy to inspect, with *mAP:0.5* values for models like Faster RCNN, FCOS, and others mostly exceeding 95%, with the lowest not falling below 85%. Conversely, defects like Damage, Dark Spot, Dirt, Flash, and Flow are more challenging to inspect. Potential reasons for this discrepancy include: a smaller number of samples for these defects, less distinct features making manual annotation errors during dataset creation more likely, and lower robustness to disturbances. PGD-net shows significant improvements in inspecting difficult defects like Damage, Dark Spot, Dirt, Flash, and Flow, with *mAP:0.5* values of 89.3%, 95.3%, 90.1%, and 89.4%, respectively. However, for Damage, the *mAP:0.5* is only 71.8%. In contrast, inspection performance is excellent for defects like Broken, Protrusion, Burn, Missing, Overflow, Perforation, Reverse, Short Shot, Void, and White, with *mAP:0.5* values all exceeding 99.1%.

In addition, this article also used a 5-fold crossover test to evaluate the generalization ability of PGD net. The mAP values for all categories in the five experiments were 95.6%, 95.1%, 95.3%, 96.0%, and 95.5%, respectively, with results all above 95.0%.

Table 5 further compares the inspection performance of different models for various defects, showcasing the inspection results for several case scenarios.

**Figure 13:**
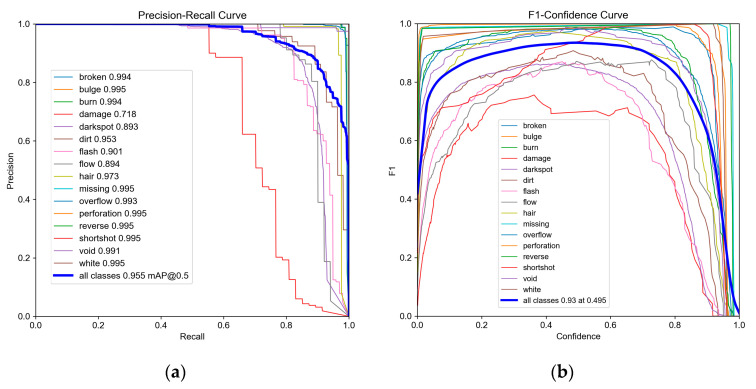
Inspection results of PGD-net for 16 types of defects. (**a**) Precision–Recall Curve; (**b**) F1–Confidence Curve.

Figure 13 presents the P–R Curve and F1–Confidence Curve of PGD-net for the 16 defect types. The P–R Curve illustrates the relationship between precision and recall at different thresholds, while the F1-score represents the harmonic mean of precision and recall, ranging from 0 to 1, with 1 being the best and 0 being the worst.

**Figure 14:**
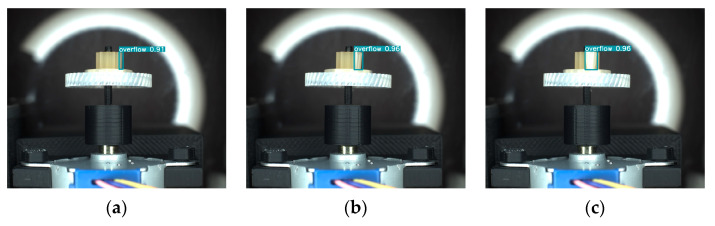

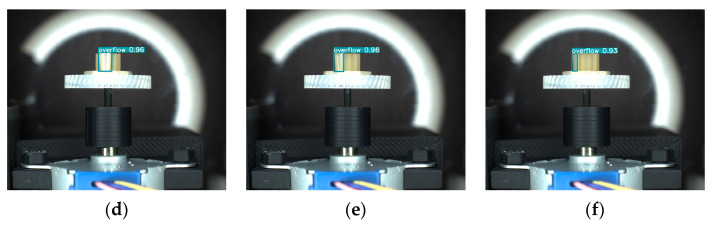
The inspection results of the same tooth surface overflow defect at different counterclockwise rotation angles. (**a**) 0 degrees; (**b**) 30 degrees; (**c**) 60 degrees; (**d**) 90 degrees; (**e**) 120 degrees; (**f**) 150 degrees.

**Figure 15:**
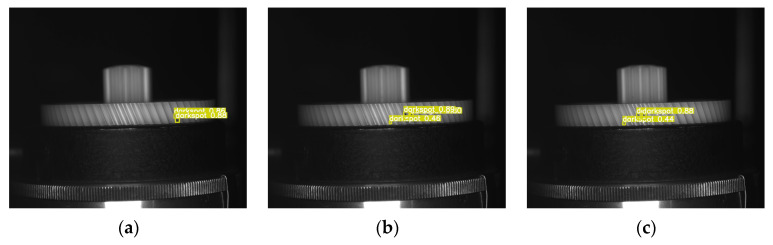
The inspection results of the same tooth surface dark spot defect at different counterclockwise rotation angles. (**a**) 0 degrees; (**b**) 30 degrees; (**c**) 60 degrees.

The tooth surface images of plastic gears are captured using a side-view camera. However, it is not possible to capture the entire tooth surface in a single image, so multiple tooth surface images need to be captured from the side. This raises a question: whether the PGD-net model proposed in this paper can inspect the same defect at different rotation angles, considering that the scale, position, and shape of the same defect may vary at different angles. To verify the inspection capability of PGD-net for the same defect at different rotation angles, side-view images were captured at different angles and subjected to inspection. The inspection results are shown in Figure 14 and Figure 15. Figure 14 depicts images captured at intervals of 30 degrees as the turntable rotates counterclockwise, resulting in a total of six images containing the Overflow defect. Figure 15 also shows images captured at intervals of 30 degrees, resulting in three images containing the Dark Spot defect. It can be observed that PGD-net can accurately inspect the same defect at different angles.

### 4.4. Focal-IoU Loss Inspection Performance Experiment

To verify the positive impact of the proposed sample weight optimization method Focal-IoU loss on inspection performance, it is compared with GIoU loss, DIoU loss, and CIoU loss in experiments. The experimental results are shown in Table 6. Among them, Focal-GIoU achieves the highest *mAP:0.5*, reaching 95.6%, which is a 0.5% improvement compared to GIoU loss. Focal-DIoU achieves an *mAP:0.5* of 95.5%, a 0.6% improvement compared to DIoU loss. Focal-CIoU also achieves an *mAP:0.5* of 95.5%, a 0.8% improvement compared to CIoU loss. The experiment demonstrates that Focal-IoU loss exhibits excellent sample balancing performance.

**Figure 16:**
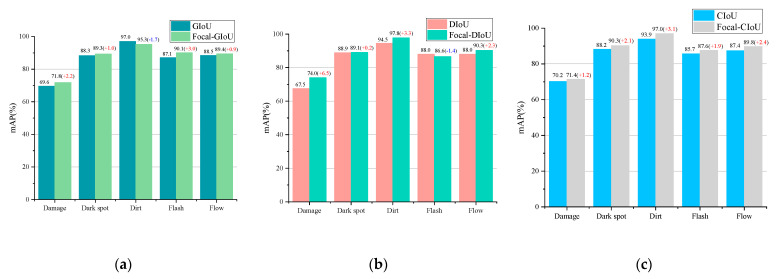
The inspection ability of Focal IoU loss for several difficult samples. (**a**) Focal-GIOU Loss vs. GIOU Loss; (**b**) Focal-DIOU Loss vs. DIOU Loss; (**c**) Focal-CIOU Loss vs. CIOU Loss.

As mentioned earlier, introducing focal weights into the IoU loss function is intended to improve the inspection accuracy of difficult samples. Several defects with previously poor inspection performance, namely Damage, Dark Spot, Dirt, Flash, and Flow, were selected as difficult samples for comparison. The results are shown in Figure 16.

Comparing Focal-GIoU loss, Focal-DIoU loss, and Focal-CIoU loss with GIoU loss, DIoU loss, and CIoU loss, respectively, reveals a significant overall improvement in inspection accuracy for several difficult defect samples. Only Focal-GIoU loss shows a decrease in inspection accuracy for the Dirt defect by 1.7%, and Focal-DIoU loss shows a decrease of 1.4% for the Flash defect. However, improvements are observed for the Damage, Dark Spot, and Flow defects. Focal-DIoU loss exhibits the highest increase in inspection accuracy for the Damage defect, with an increase of 6.5%.

### 4.5. Ablation Experiment

To validate the effectiveness of each module proposed in PGD-net, we conducted ablation experiments. Table 7 presents the results of these ablation experiments for each module.

Compared to Model A, Model E shows an improvement of 2.5% in *mAP:0.5*. With the addition of CoordConv in Model C and the incorporation of the Focal-IoU Loss module in Model D, the inference speed decreases slightly compared to Model A. However, the inference speed improves after adding the C3-FasterNetBlock module in Model E.

This suggests that the modules contribute positively to the overall performance of PGD-net, with some trade-offs in inference speed depending on the specific module.

## 5. Conclusions

This paper proposes an intelligent inspection method for comprehensive surface defects on plastic gears, termed PGD-net. In the network architecture, a sample-weight adaptive optimization method called Focal-IoU loss is designed, which alleviates the long-tail effect of defect samples on plastic gears, balances sample distribution, and enhances the inspection capability of difficult defect categories. Among these, the Focal-GIoU loss achieves the best performance, with an *mAP:0.5* of 95.6%. Moreover, replacing the ordinary convolution layers in the C3 module with FasterNet Blocks accelerates the model’s inference speed. Additionally, incorporating CoordConv in each inspection head enhances the model’s generalization ability.

Furthermore, a dataset of comprehensive surface defects on plastic gears is constructed, covering defects on the upper surface (ejector pin point surface), lower surface (point gate surface), and side surface (tooth surface), totaling 16 categories of surface defects. PGD-net achieves a comprehensive *mAP:0.5* of 95.6% for the 16 types of plastic gear surface defects, surpassing other models by at least 2.5%. The false positive rate (FPR) is 0.2%, and the false negative rate (FNR) is 6.7%. Notably, for defects such as Broken, Protrusion, Burn, Missing, Overflow, Perforation, Reverse, Short Shot, Void, and White, the *mAP:0.5* is consistently above 99.1%.

Building upon the PGD-net algorithm, this paper develops the Gear Online Inspection (GOI) system, which is deployed in plastic gear production facilities. The system features automatic feeding, automatic acquisition of gear end-face and full-tooth-face images, automatic inspection, automatic sorting and unloading, and anomaly alarm functions. It has been successfully applied in plastic gear production lines, inspecting over 60,000 gears daily.

While the constructed dataset encompasses 16 types of plastic gear surface defects, there might still be some defect types not included. Moreover, for certain complex defects, more instance images are required to expand the dataset and achieve better inspection performance. In future work, we will explore weakly supervised algorithms to inspect abnormal defects by learning from normal plastic gear sample images.

## Figures and Tables

**Table 1:** Hardware parameters of visual inspection system.

Hardware Name	Parameters
Industrial camera	Color, 3.45 μm pixel size, 2448 × 2048 resolution
Lens	Magnification 0.22×, optical distortion ≤ 0.001%
Light source	Diffuse light source

**Table 2:** Comparison of inspection results between PGD-net and other methods. Bold indicates the best value.

Model	Backbone	Epoch	mAP:0.5 (%)	Precision (%)	Recall (%)	FPR(%)	FNR(%)	CPU Infer Time (ms/piece)	Training Time (ms/piece)
Faster RCNN	Resnet-18	300	77.8	67.1	75.8	3.0	24.2	67.7	29.7
FCOS	Resnet-50	300	73.9	86.5	58.5	2.8	41.5	43.6	37.5
ATSS	Resnet-18	300	83.2	88.4	67.9	2.6	32.1	58.7	26.4
YOLOv3	Darknet-53	300	87.4	84.5	83.6	1.8	16.3	**40.4**	**19.5**
YOLOv5	Darknet-53	300	93.1	91.3	88.8	0.5	8.3	50.1	21.3
YOLOv8	Darknet-53	300	88.8	89.1	84.9	1.2	14.0	52.4	20.5
SSD	Vgg-16	300	88.3	87.1	80.9	1.9	19.1	45.1	**19.5**
PGD-net	Darknet-53	300	**95.6**	**95.0**	**92.8**	**0.2**	**6.7**	44.2	21.4

**Table 3:** The 16 types of surface defects on plastic gears.

Defect Type	Image	Remarks
Broken	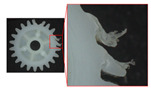	Tooth fracture or severe damage
Protrusion	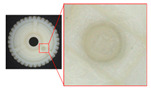	Generally occurring at the point gate
Burn	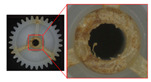	Color change and damage caused by high temperature
Damage	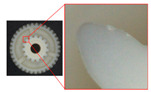	Tooth surface damage caused by collision
Dark Spot	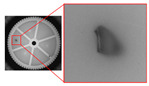	Dark impurities on the surface
Dirt	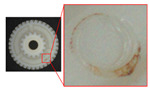	Oil stains and other dirt
Flash	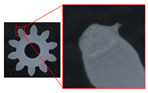	Sharp protrusions at the tooth tip, root, profile, or center hole
Flow	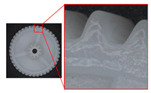	The flow traces of the formed material remain on the surface
Hair	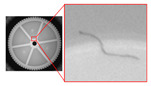	Hair adhering to the surface
Missing	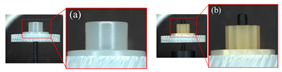	This defect is caused by the missing installation of a small tooth during secondary molding of a dual gear shown in picture (a). Acceptable product is shown in picture (b)
Overflow	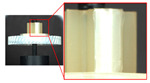	Plastic does not completely fill the mold cavity, resulting in excess adhesive material
Perforation	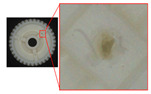	Perforation defects that generally occur at the point gate
Reverse	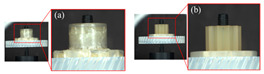	This defect is caused by the reverse installation of a small tooth during the secondary molding of a dual gear shown in picture (a). Acceptable product is shown in picture (b)
Short Shot	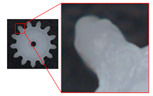	The injection molding material cannot completely fill the entire mold cavity
Void	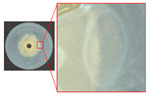	Vacuum bubbles generated by rapid freezing of material flow and obstruction of contraction
White	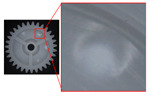	Imprints generated by the ejection rod

**Table 4:** Comparison of *mAP:0.5* (%) results for 16 defect types across different models.

Defect Type	Faster RCNN	FCOS	ATSS	YOLOv3	YOLOv5	YOLOv8	SSD	PGD-net
Broken	85.3	85.8	95.9	100	99.0	99.3	99.4	99.5
Protrusion	100	100	100	100	99.5	99.5	100	99.5
Burn	64.9	50.0	78.4	93.6	98.5	97.4	87.7	99.5
Damage	29.8	39.9	41.8	53.7	64.1	49.7	53.5	71.8
Dark Spot	54.2	72.5	61.5	69.2	84.5	74.7	68.7	89.3
Dirt	50.6	48.3	70.6	60.9	91.9	69.0	88.3	95.3
Flash	57.3	26.8	48.4	54.3	79.2	62.8	58.7	90.1
Flow	30.3	28.2	59.5	84.0	82.2	83.2	77.7	89.4
Hair	77.2	78.7	88.0	97.7	93.9	93.2	93.6	97.3
Missing	100	100	100	100	99.5	99.5	100	99.5
Overflow	92.7	91.0	95.1	93.6	97.3	97.3	94.7	99.3
Perforation	99.9	100	100	99.0	99.0	99.5	99.0	99.5
Reverse	100	100	100	100	99.5	99.5	100	99.5
Short Shot	97.6	92.9	100	99.5	99.5	99.5	100	99.5
Void	87.4	75.9	92.4	92.6	98.4	97.4	91.8	99.1
White	100	92.9	100	100	99.5	99.5	100	99.5

**Table 5:** The case comparisons of defect inspection results by various models. (Note: The colors of the inspection boxes correspond to the labels in Figure 12).

Case	Original Image	Ground Truth	Faster RCNN	YOLOv3	YOLOv5	SSD	PGD-net
Case 1	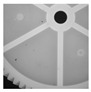	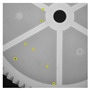	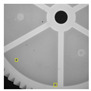	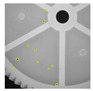	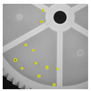	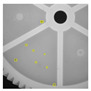	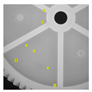
Case 2	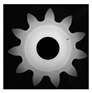	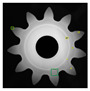	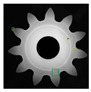	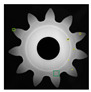	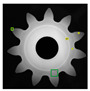	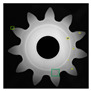	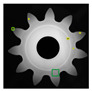
Case 3	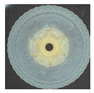	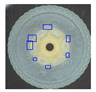	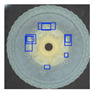	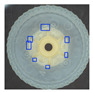	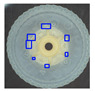	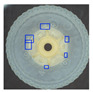	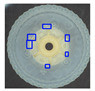
Case 4	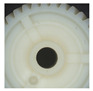	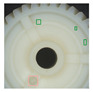	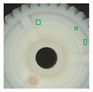	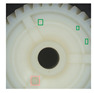	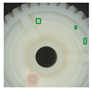	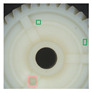	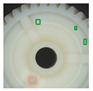
Case 5	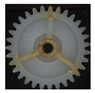	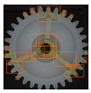	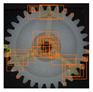	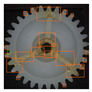	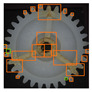	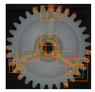	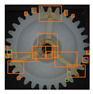
Case 6	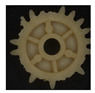	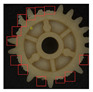	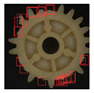	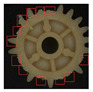	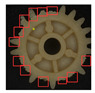	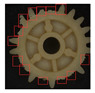	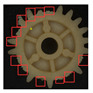
Case 7	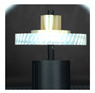	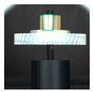	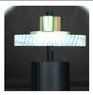	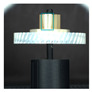	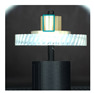	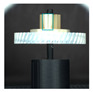	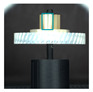
Case 8	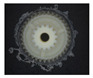	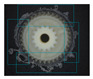	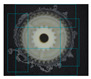	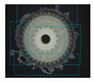	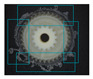	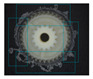	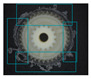
Case 9	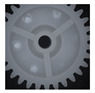	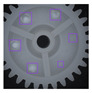	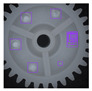	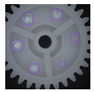	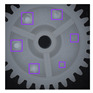	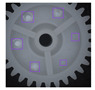	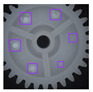
Case 10	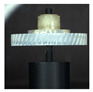	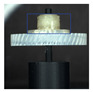	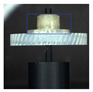	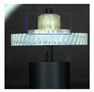	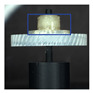	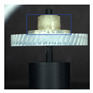	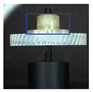

**Table 6:** The inspection performance comparison of several loss functions (γ=0.5). Bold indicates better value.

Loss Function	mAP:0.5 (%)
GIoU	95.1
Focal-GIoU	**95.6**
DIoU	94.9
Focal-DIoU	**95.5**
CIoU	94.7
Focal-CIoU	**95.5**

**Table 7:** Ablation experiment. The “√” indicates that the model has been selected for experimentation, bold indicates the best value.

Methods	C3-FasterNetBlock	CoordConv	Focal-GIoU	mAP:0.5 (%)	CPU Infer Time (ms/piece)
Model A				93.1	45.8
Model B	√			95.3	44.6
Model C		√		95.4	46.9
Model D			√	95.6	45.9
Model E	√	√	√	95.6	45.6

## Data Availability

The data that support the findings of this study are available from the authors upon reasonable request.
